# Variations in advertisement call modulations do not influence vocal interactions in bullfrog choruses

**DOI:** 10.1121/10.0015070

**Published:** 2022-11-07

**Authors:** Andrea Megela Simmons, Laura N. Kloepper

**Affiliations:** 1 Department of Cognitive, Linguistic and Psychological Sciences, Brown University, Providence, Rhode Island 02912, USA; 2 Department of Biological Sciences, University of New Hampshire, Durham, New Hampshire 03824, USA Andrea_Simmons@brown.edu, Laura.Kloepper@unh.edu

## Abstract

Chorusing male bullfrogs naturally vary the number of modulations within their advertisement call notes. A field playback experiment investigated whether these variations affect males' evoked vocal responses. Vocal responses were quantified manually and automatically by quantifying acoustic energy. The numbers of calls, number of notes, latency of response, and detected-note acoustic energy did not vary significantly across playback stimuli for focal males or the entire chorus, suggesting that variations in modulation number do not carry relevant information to males. Future work can determine whether modulation cues may function in sexual selection and affect female response.

## 1. Introduction

Anuran amphibians are highly vocal animals whose reproductive behaviors depend largely on recognition and localization of acoustic cues. Male anurans produce species-specific advertisement (mating) signals that function for mate attraction and warning rivals (Gerhardt, 1994). An important question for bioacoustic analysis is understanding what acoustic features of these signals are most salient for different receivers (males or females), which features are stereotyped, which features are variable and inconsistent, and which features may be present but irrelevant for communication (Gerhardt, 1994; Gerhardt and Bee, 2007; Reichart, 2013; Tanner and Bee, 2019, 2020). We add to this literature by investigating how variations in a prominent feature of the male bullfrog's (*Rana catesbeiana*) advertisement call, the number and patterning of modulations in individual call notes, influence evoked vocal responses by rival chorusing males.

**Fig. 1.**
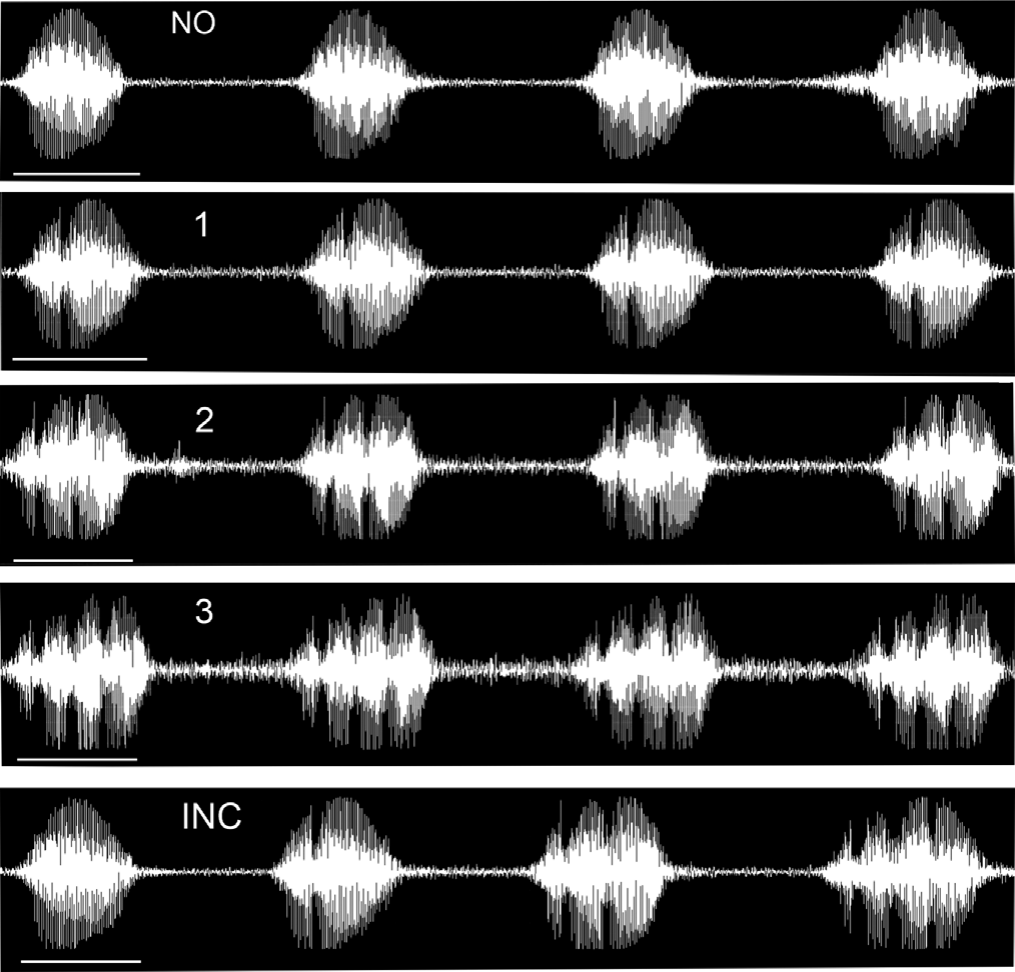
Playback stimuli. Notes were digitized from a field-recorded natural advertisement call and then rearranged to form the five four-note patterns illustrated. The numbers of modulations in each stimulus are indicated at the top of each plot. NO = no note modulations (representative of natural calls). Stimulus 1 (each note contains one modulation), stimulus 2 (each note contains two modulations), and stimulus 3 (each note contains three modulations) are not representative of the patterns of note modulation seen in natural calls, although they do contain natural numbers of modulation. INC = each note has an increasing number of modulations (representative of natural calls). Durations of individual notes (480–595 ms), internote intervals (500–530 ms), and overall stimulus duration (3.8–4.2 s) are all within the natural range (Capranica, 1965; Bee, 2004; Simmons, 2004). Waveforms were made in Adobe Audition with a sampling rate of 8000 Hz, 16-bit.

The male bullfrog's advertisement call consists of 2–12 multiple-harmonic notes (Capranica, 1965; Bee, 2004). The call's frequency spectrum, harmonic structure, periodicity, and duration of individual notes are important cues mediating male-male vocal interactions within choruses (Capranica, 1965; Davis, 1987; Simmons and Bean, 2000; Bee and Gerhardt, 2001; Simmons, 2004; Gerhardt and Bee, 2007; Bee *et al.*, 2016). Suggs and Simmons (2005) described a prominent acoustic feature of this call whose function is unknown. When male bullfrogs vocalize a series of notes, they progressively vary the shape of the envelope of successive notes from smooth and unmodulated to rough and modulated (Fig. 1). These changes were described as variation in the number of modulations or “gaps” (as perceived by human listeners) within each note. Three simple rules explain the variations observed in natural calling. First, all of the calls begin with an unmodulated note. Second, modulations begin with the addition of one modulation. Third, once modulations begin, their number varies across successive notes with an increase or a decrease of one modulation only. The regularity of this pattern suggests that modulations serve some biological significance in the communication behavior of the bullfrog, but this proposal has not been tested in either male or female receivers.

We undertook an evoked calling playback experiment to explore how these natural changes in note modulation number might affect male-male vocal interactions. We identified a unique focal male in a chorus and quantified his responses to playbacks of advertisement call notes varying in numbers of modulations. We also quantified the evoked responses of the entire chorus to the playback stimuli. Our rationale for this chorus-level analysis is based on observations that male bullfrogs call preferentially in response to far, opposed to nearby, neighbors (Boatright-Horowitz *et al.*, 2000; Bates *et al.*, 2010). Thus, a male far away from the sound source may be the first to respond to an advertisement call by either a real or simulated caller, triggering calls by the rest of the chorus, including those closer to the sound source. Quantifying the evoked vocal response of only one focal male may not capture the effectiveness of a particular playback stimulus for mediating vocal interactions within the group. In addition, male bullfrogs often emit notes synchronously or in patterns of note-by-note alternation with other males (Bates *et al.*, 2010). Separating out notes from individual males can be challenging in a focal male analysis but not in a chorus-level analysis where calls from all active males are considered.

As part of the chorus-level analysis, we tested the effectiveness of an analysis method based on quantification of total acoustic energy for use in playback experiments. This method was first introduced by Kloepper *et al.* (2016) for estimating population sizes of swarming bats and adapted by Simmons *et al.* (2022) for monitoring spontaneous vocal activity in small bullfrog choruses. Measures of total acoustic energy can provide an estimate of aggregate vocal activity without the time-consuming need to identify calls of individual males.

We hypothesized that male bullfrogs would respond more to playback stimuli that are representative of a natural sequence of variations in modulation number of successive notes. On the other hand, if these variations have no communicative significance for rival chorusing males, then numbers of evoked vocal responses or response acoustic energy should not vary between stimuli.

## 2. Methods

### 2.1 Recording sites

Bullfrogs were not handled or captured for these experiments. Playbacks were performed on six nights at two natural ponds containing small bullfrog choruses. At site 1, calling spots of actively vocalizing males (4, 5, or 6 on different nights) were separated by 3–15 m. We identified five actively vocalizing males on two recording nights at site 2 with calling spots separated by 6–18 m. We made no assumptions whether the same males participated in the chorus on subsequent nights at the same location.

### 2.2 Playback stimuli

Playback stimuli were created from archival natural advertisement calls of an individual male bullfrog. Calls from this male had excellent signal-to-noise ratios with spectral and temporal parameters near the population mean (Bee, 2004). We chose examples of calls with no modulations and examples with variable numbers of modulations. These natural calls were digitized and converted to 16 000 Hz 16-bit .wav format using CoolEdit 2000 (Syntrillium Software, Phoenix, AZ). Individual notes in the digitized calls were then rearranged to construct the five four-note stimuli appearing in Fig. 1. Stimulus NO (no modulations) and stimulus INC (increasing numbers of modulations) represent patterns present in field-recorded natural calls. Stimuli 1, 2, and 3 were constructed by repeating one note (1, one modulation; 2, two modulations; 3, three modulations, respectively) for the entire four-note call. We did not construct stimuli with more than three modulations as these are rare in natural recordings. In natural calls, increasing numbers of modulations co-occur with increases in note duration (Suggs and Simmons, 2005). Our playback stimuli mirror this pattern with individual notes increasing in duration from 480 to 595 ms as the number of modulations increases. Previous work showed that male bullfrogs do not respond differentially to these changes in note duration (Simmons, 2004).

All 5 stimuli were organized into 3 different random orders of 30 stimuli each (6 repetitions of each of the 5 stimuli) with interstimulus intervals of 20 s within the natural range (Boatright-Horowitz *et al.*, 2000; Bee, 2004). Stimuli were recorded on Sony digitial audio tapes for subsequent playback in the field.

### 2.3 Experimental procedure

Playback experiments were conducted on four nights (two repetitions, each with a different stimulus order, separated by 20 min on two of these four nights; only one repetition on two other nights) at site 1 and two nights (two repetitions separated by 20 min on one night, one repetition on the other night) at site 2, all were between 21.00 h and 24.00 h. Air and water temperatures ranged between 21 °C and 26 °C.

Stimulus tapes were played through a Sony TCD-D8 tape recorder (Sony, New York) and an amplifier-speaker (Pignose Hog30 Model 7-3000, Pignose-Gorilla, Las Vegas, NV) positioned at the water's edge, facing out toward the center of the pond. The stimulus level was calibrated to 80 dB sound pressure level (SPL; re 20 *μ*Pa) at 1 m, the mean amplitude of natural advertisement calls (Megela-Simmons, 1984), using a Realistic sound level meter (flat weighting). A Sennheiser ME66 microphone (frequency response 0.05–20 kHz, ±3 dB; Sennheiser, Wennebostel, Germany) was positioned on a Styrofoam platform and placed in the water approximately 1 m from a focal male. Focal males were identified based on strong calling activity during baseline listening, by distance to the speaker (ranging from 6 to 12 m) and by distance to each other (calling spots separated by 12–15 m). A different focal male was chosen for each playback session. Activity of the entire chorus was recorded simultaneously. Response vocalizations were routed to one channel of a Marantz model PMD430 cassette recorder (frequency response 50–14 000 Hz, ±3 dB; Marantz, Mahwah, NJ). The line output of the Sony recorder was recorded onto the second channel as a stimulus marker. Onset of playback tapes was triggered manually. If any male in the chorus continued to vocalize near the upcoming end of the 20 s interstimulus interval, we manually stopped the presentations to avoid overlap with the next stimulus presentation. This resulted in actual interstimulus intervals of up to 40 s, which are, again, within the natural range.

### 2.4 Data analysis

Audio recordings were digitized as .wav files at 16 000 Hz, 16-bit using Adobe Audition (Adobe, San Jose, CA). These files were then segmented using the time selection tool in Adobe Audition into five stimulus-specific files (one for each stimulus, each file containing six repetitions of that stimulus on each playback session). For manual analysis, we tallied the number of advertisement calls and the number of individual advertisement call notes evoked from (1) each focal male in that playback session and (2) all males who vocalized in the interstimulus interval, regardless of whether they were the first or subsequent male to respond and regardless of their distance from the recording microphone. Latency of response from the onset of the first note in the stimulus to the onset of the first note from the first responding male was also measured. Manual counts of responses (manually detected notes and calls) were made by ear and visual inspection of spectrograms (spectral frequency display in Adobe Audition). Notes emitted in the presence of background noise or those from frogs far from the recording microphone were included as long as the spectrograms indicated clearly the presence of the appropriate harmonic spectrum. One note calls, which may function in an aggressive context (Capranica, 1965; Davis, 1987; Bee *et al.*, 2016), were excluded from analyses because of their low and inconsistent prevalence (mean 4.8% of all calls over all nights; range, 0%–6.7%).

For automated identification of call notes, we identified segments of each digitized .wav file containing anthropogenic noise to construct a noise print in Adobe Audition using the noise reduction algorithm. This noise print was then applied to that entire stimulus-specific .wav file to reduce this interfering noise. Next, we manually cut the playback stimuli from each of the .wav files using the time selection tool; because of the spatial arrangement of males in the chorus and the playback level of 80 dB SPL, it was not possible to avoid having the stimulus picked up by the recording microphone. Care was taken to retain any answering notes that occurred in the time intervals between the four notes in the playback stimulus; however, any answering notes that overlapped playback notes were lost in this process.

A custom matlab 2021a (The MathWorks, Natick, MA) script (based on Kloepper *et al.*, 2016) was developed to calculate numbers of automatically detected notes and detected-note acoustic energy. The algorithm is described fully in Simmons *et al.* (2022). Prior to the energy summation, the noise-reduced .wav files were passed through a Butterworth filter with a cutoff frequency of 2000 Hz and then an equiripple finite impulse response (FIR) filter (passband ripple, 0.5 dB; stop band attenuation, 65 dB at 1 and 2.5 kHz). The distribution of the amplitudes in the filtered waveform was fit with a normal function, and a level of twice the standard deviation of the amplitude was used as a threshold for determining peaks (i.e., peak amplitude of individual or overlapping notes) in the waveform. A bounding box was calculated around these peaks (widths of 0.2–2 s) so as to capture the detected-note acoustic energy (*V*^2^) in single or overlapping bullfrog notes while excluding other sound sources.

All of the statistics on focal male and chorus-level data were conducted in SPSS Statistics v. 27 (IBM, Armonk, NY). We used nonparametric repeated measures Friedman tests (two-tailed) to compare the number of answering calls, number of answering notes, and latency of first response across playback stimuli. We used a Pearson correlation coefficient to correlate the number of manually detected notes to automatically detected notes. Detected-note acoustic energy and its relationship to manually detected notes were analyzed only in chorus-level data.

## 3. Results

### 3.1 Focal male analyses

**Fig. 2.**
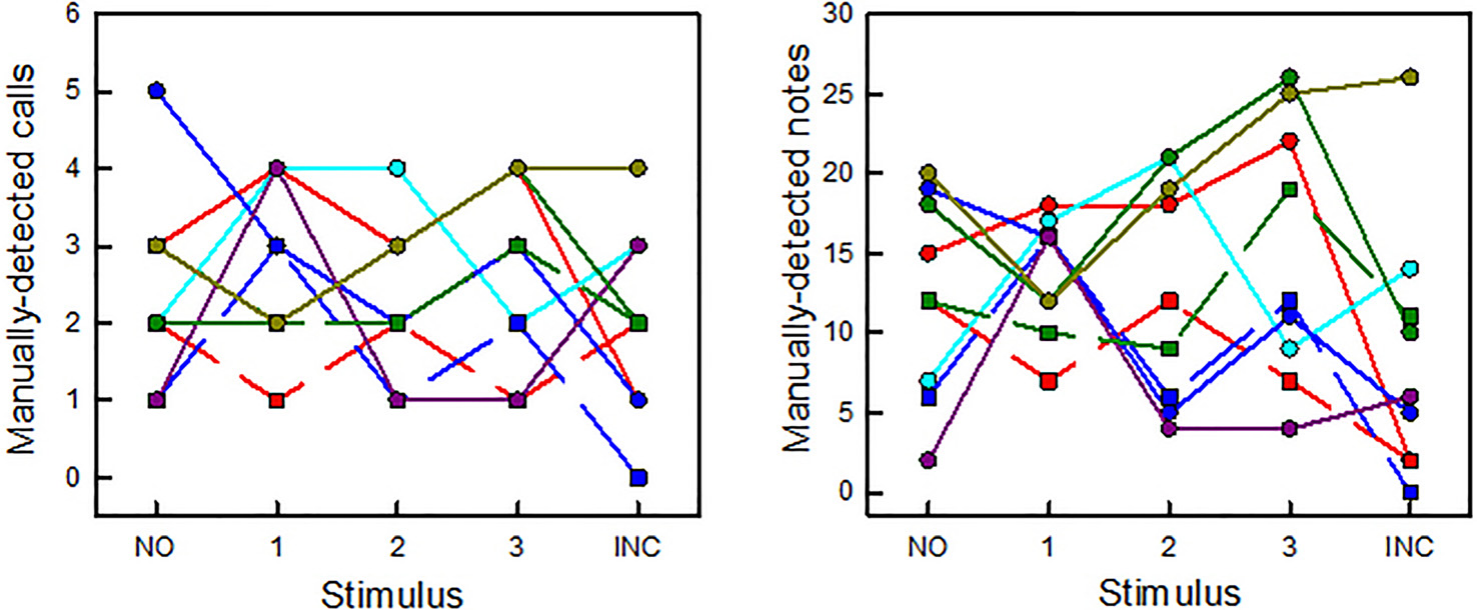
The total numbers of answering calls (left) and answering notes (right) given by nine focal males to each playback stimulus. A different focal male was identified in each playback session, and no male was tested more than once. Data are based on manual counts and plotted individually for each male using a unique color symbol and line. Males recorded on the same night in two different sessions are represented by the same color, but different symbol/line shape (i.e., red circle, solid and red square, dashed). There are no significant differences in responses across the five playback stimuli (see the text).

Vocal activity by nine individual focal males was quantified. Numbers of manually detected calls and numbers of manually detected notes emitted by these nine males in response to the five playback stimuli did not vary statistically [calls, *X*^2^ = 2.16, degrees of freedom (df) = 4, *p* = 0.71; notes, *X*^2^ = 3.84, df = 4, *p* = 0.43; Fig. 2]. Latency of the focal male's response also did not vary (*X*^2^ = 1.35, df = 4, *p* = 0.85).

### 3.2 Chorus-level analyses and acoustic energy measures

**Fig. 3.**
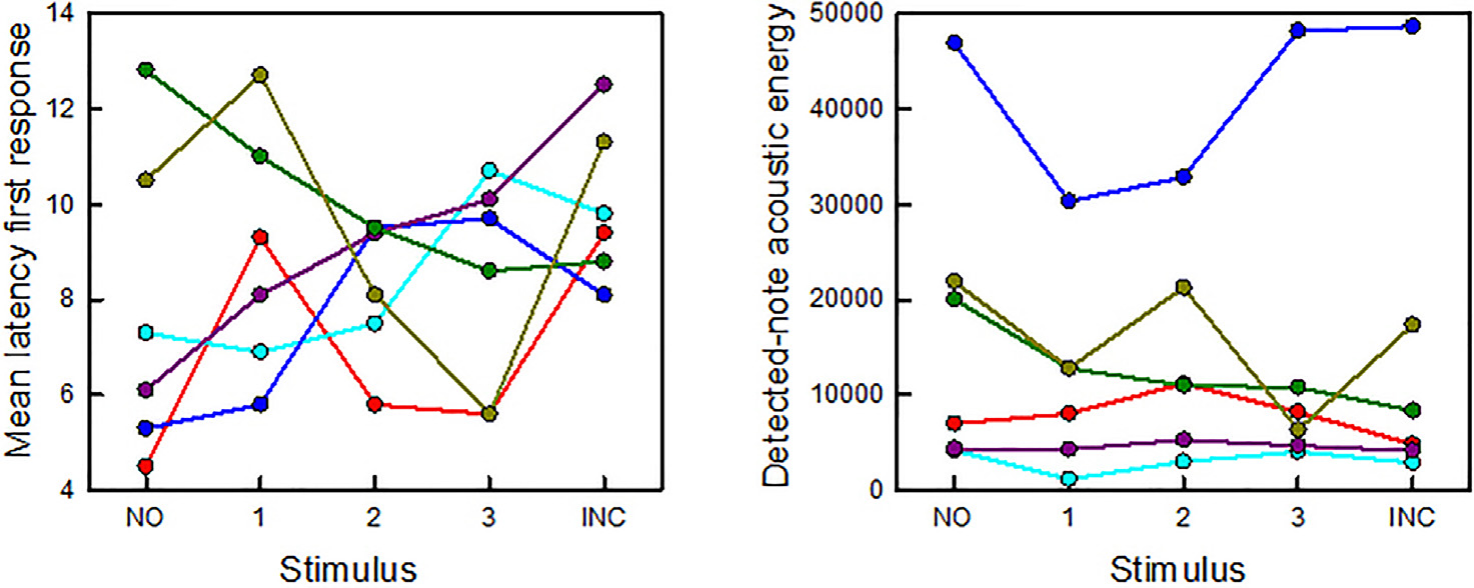
The results of chorus-level analyses. Latency of response to the playback stimuli (left) was measured manually from the onset of the stimulus to the onset of the first note emitted by the first responding male in the chorus. Data are plotted as the mean latency over six repetitions of each stimulus for that playback session. Detected-note acoustic energy (right), as quantified automatically, is also plotted as the mean for that playback session. Each color symbol/line corresponds to the individual male data shown in Fig. 2. There are no significant differences in latency or detected-note acoustic energy across the five stimuli (see the text).

For those three recording nights in which playbacks were conducted twice, we averaged the data from the two repetitions so as to avoid pseudoreplication. On these nights, chorus size remained the same for the two repetitions. A total of six choruses was used for analysis. There was no significant difference in numbers of answering notes (manually detected) by the chorus across the five playback stimuli (*X*^2^ = 2.89, df = 4, *p* = 0.58). Latency of the first note emitted by any responding male did not vary significantly across stimuli (*X*^2^ = 2.36, df = 4, *p* = 0.67; Fig. 3, left). The detected-note acoustic energy (*X*^2^ = 6.1, df = 4, *p* = 0.19; Fig. 3, right) did not vary significantly across playback stimuli.

**Fig. 4.**
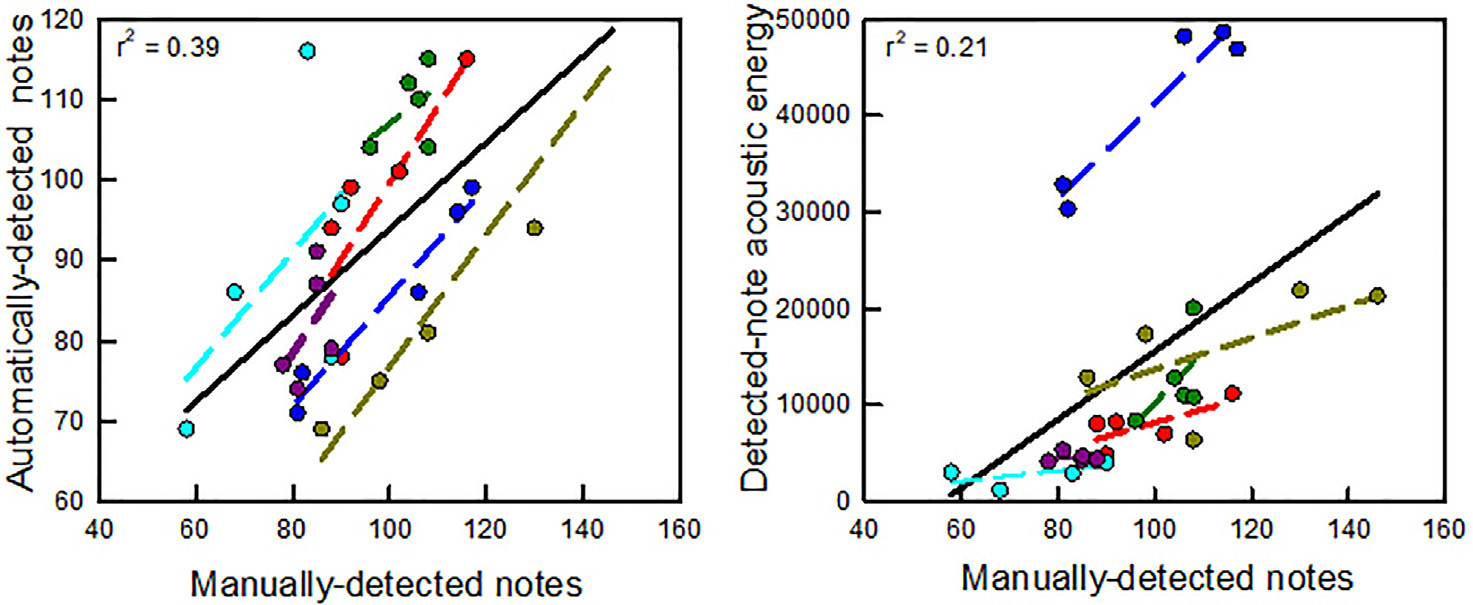
(Left) Regressions between counts of automatically detected notes and manually detected notes. The solid black line and the *r*^2^ value (top left inset) show results from all six playback sessions combined. There is a positive relationship between automatically detected and manually detected notes. Regressions calculated from the data for each playback session are shown by colored symbols (five for each color, representing the five stimuli) and colored dashed lines, using the same convention and male identification as in Fig. 3. Individual *r*^2^ values are 0.68 (red), 0.95 (blue), 0.22 (dark green), 0.03 (cyan), 0.24 (purple), and 0.93 (brown). (Right) Regressions between detected-note acoustic energy and counts of manually detected notes. The solid black line and *r*^2^ value (top left inset) show results from all of the playback sessions combined. Regressions for each session are shown by the same colored symbols and colored dashed lines as in the left plot. Individual *r*^2^ values are 0.51 (red), 0.91 (blue), 0.36 (dark green), 0.39 (cyan), 0.005 (purple), and 0.38 (brown).

The automated algorithm demonstrated good performance compared to manual extraction. Across all of the recording nights, both notes and acoustic energy between the manually detected and automatically detected approaches were significantly correlated (notes, Pearson correlation coefficient, *r *=* *0.62, *n* = 30, *p* < 0.001; *r*^2^ = 0.39, Fig. 4, left; energy, *r *=* *0.46, *n* = 30, *p* = 0.01; *r*^2^ = 0.21; Fig. 4, right). Correlations between manually detected and automatically detected notes on individual nights ranged from 0.03 to 0.95. For most nights, correlations between manually detected notes and detected-note energy ranged between 0.36 and 0.91 with one night's recording having a low range of manually detected notes and resulting low correlation (purple line, Fig. 4, right).

## 4. Discussion

Acoustic cues play important roles in assessment and recognition of rivals in anuran choruses, but identifying which cues are critical and which are not has been examined in males of relatively few species (Bee *et al.*, 2016). Our data show that a prominent acoustic feature of male bullfrog advertisement calls, variation in envelope modulation number across successive notes, is likely irrelevant (Gerhardt and Bee, 2007) for male-male communication.

If note modulation number played a role in male-male competition for females, we would expect to find significant changes in males' vocal responses across the different playback stimuli. This expectation was not borne out by our data. We found no significant differences in number of manually detected notes, automatically detected notes, latency of first response, or detected-note acoustic energy across stimuli varying in note modulation number. This lack of differential response held true even for modulation patterns (stimuli 1, 2, and 3) that do not represent a natural pattern in advertisement calls even though the numbers of modulations within these stimuli are themselves within the natural range. Modulations may, on the other hand, provide important cues for female mate choice. Future playback experiments can investigate whether females respond differentially to variations in note modulations within the range that males naturally produce. It is possible that envelope modulations reflect variations in and limitations on the muscular control involved in vocal production (Schmidt, 1965; Walkowiak, 2006) rather than playing some communicative function for either males or females. This is a subject for biomechanical investigation.

Another important outcome of this study is the comparison between automated and manual call analysis. Manual quantification of evoked vocal responses can be time-consuming, especially when studying dense or multi-species choruses, and requires trained listeners. Methods based on automated detection and machine learning can overcome the limitations of manual analyses (Yen and Fu, 2001; Waddle *et al.*, 2009; Herrick *et al.*, 2018). Our work is further validation that automated call approaches can be useful for quantifying vocal responses from anurans in the field. Our algorithm for automated detection of notes and calculating acoustic energy correlated well with the manual analyses. Correlations between manual and automated approaches were highest for sessions with the greatest range of manually detected notes (i.e., all except the purple session in Fig. 4, right), and manual detection performed better in detecting low-amplitude calls that did not exceed our detection threshold. Because the automated approach cannot distinguish between individuals vocalizing at different distances, microphone arrays would likely greatly improve the performance of our automated analysis.

It is important to address some of the methodological limitations with our study. First, it is possible that males modify their vocal response to stimuli containing different note modulations in ways other than those we attempted to quantify. Although the variations in note modulation we examined here had no influence on male responding, they may be important in conjunction with other acoustic features (Ryan and Rand, 2003; Gerhardt and Bee, 2007). Second, like many field experiments, small sample size limits the statistical power of this analysis. Data from a subsequent field season (see the supplementary material,^1^ Fig. 1), which presented only three of the five playback stimuli used in this experiment, confirmed that eight new focal males did not distinguish between stimuli varying in number of note modulations by their evoked calling responses. Third, we used only one exemplar for our playback study. It is possible that a different stimulus with different acoustic properties would evoke a different pattern of response; therefore, expanding future work to include playback examples from more than one individual would more strongly support our conclusions. Finally, because we tested only modulation numbers within the range observed in natural calls (Suggs and Simmons, 2005), our experiment does not address whether modulations outside this biological range would elicit differential responses.

## Footnotes

^1^
See supplementary material at https://www.scitation.org/doi/suppl/10.1121/10.0015070 for results of a related experiment.
